# Brassinosteroids are involved in Fe homeostasis in rice (*Oryza sativa* L.)

**DOI:** 10.1093/jxb/erv079

**Published:** 2015-03-14

**Authors:** Baolan Wang, Gen Li, Wen-Hao Zhang

**Affiliations:** ^1^ State Key Laboratory of Vegetation and Environmental Change, Institute of Botany, the Chinese Academy of Sciences, Beijing 100093, P. R. China; ^2^ Research Network of Global Change Biology, Beijing Institutes of Life Sciences, Chinese Academy of Sciences, Beijing, P. R. China

**Keywords:** Brassinosteroids (BRs), *d2-1* mutant, Fe deficiency, Fe translocation, Fe uptake, rice (*Oryza sativa*), strategy II plant.

## Abstract

Brassinosteroids (BRs) are steroid hormones that modulate numerous physiological processes in plants. However, few studies have focused on the involvement of BRs in sensing and responding to the stress of mineral nutrient deficiency. In the present study, we evaluated the roles of BRs in the response of rice (*Oryza sativa*) to iron (Fe) deficiency during Fe uptake, transport, and translocation. Exogenous application of 24-epibrassinolide (EBR) to wild-type (WT) plants exaggerated leaf symptoms of Fe deficiency and suppressed growth. EBR increased and decreased Fe concentrations in roots and shoots, respectively, under both Fe-deficient and Fe-sufficient conditions. Transcripts involved in Fe homeostasis, including Os*IRT1*, Os*YSL15*, Os*YSL2*, Os*NAS1*, and Os*NAS2*, were enhanced by EBR under Fe-deficient conditions. EBR depressed expression of Os*NAS1*, Os*NAS2*, and Os*YSL2* in shoots, and inhibited Fe transport and translocation via the phloem. Rice mutant *d2-1*, which is defective in BR biosynthesis, was more tolerant to Fe deficiency than the WT, and accumulated greater amounts of Fe in roots than the WT under Fe-sufficient conditions. A greater upregulation of Os*IRT1*, Os*YSL15*, Os*YSL2*, Os*NAS1*, and Os*NAS2* in the *d2-1* mutant compared to the WT was found under Fe-sufficient conditions, while expression of these genes in the *d2-1* mutant was lower than in the WT under Fe-deficient conditions. The greater tolerance of the *d2-1* mutant could be partly mitigated by exogenous application of EBR. These novel findings highlight the important role of BR in mediating the response of strategy II plants to Fe deficiency by regulating Fe uptake and translocation in rice.

## Highlight

Brassinosteroids negatively regulate Fe transport and translocation in a strategy II rice plants.

## Introduction

Iron (Fe) is one of the essential mineral nutrients required for plant growth and development. Plant growth is often limited by low availability of Fe in soil due to its low solubility, especially in calcareous soils (Romera and Alcántara, 2004). Plants have evolved various strategies to cope with Fe deficiency in soil. These strategies have been classiﬁed as strategy I and strategy II, and these exist in non-graminaceous monocots and dicots, and in graminaceous monocots, respectively (Römheld and Marschner, 1986; Kobayashi and Nishizawa, 2012). Increased ferric chelate reductase activity, acidification of the rhizosphere, and upregulation of Fe^2+^ transporters (IRTs) are major mechanisms by which strategy I plants maximize their Fe acquisition (Curie and Briat, 2003; Hell and Stephan, 2003; Romera and Alcántara, 2004). In contrast, strategy II plants commonly secrete phytosiderophores (PSs) belonging to the mugineic acid (MA) family from roots to solubilize Fe^3+^ (Kobayashi and Nishizawa, 2012). A gene encoding an Fe^3+^-MA transporter has been isolated from maize mutant *yellow trip 1*, and an Fe^3+^-phytosiderophore uptake transporter was named as Yellow Stripe1 (YS1) (von Wiren *et al.*, 1994; Curie *et al.*, 2001). Secretion of MAs from rice roots to the rhizosphere is mediated by Os*TOM1* (Nozoye *et al.*, 2011), and the resulting Fe^3+^-MA complexes are adsorbed into root cells by yellow-stripe like (YSL) transporters in the plasma membrane (Conte and Walker, 2011). In rice, OsYSL15 is the primary transporter responsible for uptake of Fe^3+^-MA from the rhizosphere (Inoue *et al.*, 2009). Recent studies reveal that, in addition to acquisition of Fe by MA, rice plants also possess the Fe^2+^-transporter system, and two genes encoding Fe^2+^ transporters, Os*IRT1* and Os*IRT2*, have been isolated (Bughio *et al.*, 2002; Ishimaru *et al.*, 2006). Expression of Os*IRT1* and O*sIRT2* is upregulated by Fe deficiency, and mutation in NAAT stimulates the Fe (II) acquisition system in cultures with an abundant Fe^2+^ source, leading to Fe accumulation in rice plants (Cheng *et al.*, 2007).

Iron is transported in complexed forms once it is loaded into the xylem (Conte and Walker, 2011). In *Arabidopsis*, FRD3, which belongs to the multidrug and toxic compound extrusion (MATE) family, is involved in loading of citrate into the xylem (Rogers and Guerinot, 2002; Green and Rogers, 2004; Durrett *et al.*, 2007). *FRD3* is expressed in the root vasculature, and its expression is enhanced by Fe deficiency (Green and Rogers, 2004). Similar to *Arabidopsis*, rice also possesses an *FRD*-like gene (Os*FRDL1*), which encodes a citrate transporter localized at the rice root pericycle cells and mediates the translocation of Fe to the shoot in the form of Fe-citrate complex (Yokosho *et al.*, 2009). A recent study reported that Fe exists as oxo-bridged tri-Fe^3+^, tri-citrate (Fe_3_Cit_3_) complex in the xylem sap of Fe-deficient tomato (Rellán-Álvarez *et al.*, 2010). In addition to citrate, nicotianamine (NA) is another important agent to complex metals in plants. Nicotianamine exists ubiquitously in roots and shoots as well as in the xylem and phloem sap of higher plants, and it can complex Fe^2+^, Fe^3+^, Mg^2+^, and Zn^2+^ (Morrissey and Guerinot, 2009; Conte and Walker, 2011). In rice, nicotianamine is synthesized by NA synthase (NAS) from *S*-adenosylmethionine, and involves long-distance Fe transport (Inoue *et al.*, 2003). OsYSL2 is a rice metal-NA transporter responsible for translocation of Fe and Mn into the grain via the phloem (Koike *et al.*, 2004). Moreover, OsYSL2 can also play roles in the mediation of translocation of Fe from root to shoot (Ishimaru *et al.*, 2010).

A number of phytohormones and messenger molecules such as auxin, ethylene, cytokinins, and nitric oxide (NO) have been reported to be involved in the regulation of Fe deﬁciency-induced changes in morphological and physiological processes in strategy I plants (Ivanov *et al.*, 2012). In contrast, there is limited information on the role of phytohormones in the response of strategy II plants to Fe deficiency (Wu *et al.*, 2011). As a class of plant polyhydroxysteroids, brassinosteroids (BRs) are ubiquitous in plants (Noguchi *et al.*, 1999). There is emerging evidence demonstrating that BRs play important roles in the response of plants to biotic and abiotic stresses (Sasse, 2003). Our previous studies showed that brassinosteroids are involved in response of cucumber (*Cucumis sativus*) to Fe deﬁciency by regulating Fe deﬁciency-induced FRO and Fe translocation from roots to shoots (Wang *et al.*, 2012). In the present study, we evaluated the role of BRs in the response of strategy II rice plants to Fe deficiency using wild-type rice and the BR-deficient rice mutant *d2-1*. The *d2-1* plants are dwarf mutants resulting from a defect in BR biosynthesis (Hong *et al.*, 2003). The *D2* gene encodes a novel cytochrome P450 classified as CYP90D that is highly similar to the reported BR synthesis enzymes. The D2/CYP90D2 enzyme catalyses the steps from 6-deoxoteasterone to 3-dehydro-6-deoxoteasterone and from teasterone to 3-dehydroteasterone in the late BR biosynthesis pathways (Hong *et al.*, 2003). Our results demonstrate that BR also played an important role in the response of strategy II plant to Fe deficiency by regulating long-distance transport and translocation of Fe via the phloem.

## Materials and methods

Wild-type (WT) rice (*Oryza sativa* L. cv. ‘Taichung 65’) and *d2-1* mutants derived from Taichung 65 were used. The *d2-1* plants were screened from a mutant library produced by *N*-methyl-*N*-nitrosourea. Hong *et al.* (2003) cloned the *D2* gene by map-based cloning and showed quantitatively that endogenous BR levels in the mutants were much lower than in their counterpart WT plants under normal growth conditions. The seeds were surface sterilized by incubation for 3min in 75% ethanol followed by 10min in 0.1% HgCl_2_, and were then washed thoroughly with sterile water. The sterilized seeds were soaked in water for 24h in the dark and then transferred to a nylon net floating on water for 1 week. Thereafter, the 7-d-old seedlings were transferred to nutrient solution containing half-strength Kimura B solution. The nutrient solution contained the macronutrients (mM) (NH_4_)_2_SO_4_ (0.18), MgSO_4_·7H_2_O (0.27), KNO_3_ (0.09), Ca (NO_3_)_2_·4H_2_O (0.18), and KH_2_PO_4_ (0.09); and micronutrients (µM) MnCl_2_·4H_2_O (0.5), H_3_BO_3_ (3), (NH_4_)_6_Mo_7_O_24_·4H_2_O (1), ZnSO_4_·7H_2_O (0.4), and CuSO_4_·5H_2_O (0.2). The solution also contained 50 µM FeEDTA. The pH was adjusted daily to 5.5 and the solution was renewed every 3 d. The hydroponic experiments were carried out in a growth room with a 16-h light (30°C)/8-h dark (22°C) photoperiod, and the relative humidity was kept at ~70%. After 10-d growth, the pre-cultured seedlings were used for the following experiments.

### Measurement of chlorophyll

Ten-d-old WT seedlings pre-cultured in solution containing 50 µM Fe-EDTA were transferred to full-strength Kimura B solution supplemented with 0 µM (Fe-deficient medium) or 100 µM Fe-EDTA (Fe-sufficient medium) with varying concentrations of 24-epibrassinolide (EBR) (0, 1, 10, 100, and 500nM) for 2 weeks. The EBR was dissolved in a minimal volume of ethanol, and then made up to volume with nutrient solution (Yu *et al.*, 2004). After treatment, foliar chlorophyll concentrations were measured. Newly formed leaves were weighed and then ground with aqueous acetone (80% v/v) and centrifuged at 10 000*g* for 5min. Absorbance (A) readings of the supernatant were recorded at 645 and 663nm. Total chlorophyll concentration (mg l^–1^) was calculated as 8.02A663 + 20.21A645, and expressed as mg chlorophyll g^–1^ fresh weight. Ten-d-old WT and *d2-1* mutant seedlings pre-cultured in solution containing 50 µM Fe-EDTA were transferred to full-strength Kimura B solution supplemented with 0 µM (Fe-deficient medium) or 100 µM Fe-EDTA (Fe-sufficient medium) with or without 100nM EBR for 2 weeks. Foliar chlorophyll concentrations were measured.

### Determination of plant biomass and metal analysis in roots and shoots

Ten-d-old WT and *d2-1* seedlings pre-cultured in solution containing 50 µM Fe-EDTA were transferred to 0 µM (–Fe) and 100 µM (+Fe) Fe-EDTA full-strength Kimura B solution in the absence and presence of 100nM EBR for 2 weeks. After treatments, seedlings were harvested and separated into roots and shoots, then dried for 2 d at 75ºC. After measurements of biomass, root and shoot samples were ground to ﬁne powder and digested with concentrated nitric acid and hydrogen peroxide. The total Fe concentrations were determined by Inductive Coupled Plasma Emission Spectrometry (ICP-OES).

### Expression patterns of genes encoding Fe uptake and translocation proteins

Total RNA was extracted from roots and leaves of rice plants subjected to Fe or EBR treatments for varying periods (1, 3, and 7 d). Total RNA was isolated using RNAiso reagent (Takara) and reverse-transcribed into first-strand cDNA with PrimeScript® RT reagent Kit with gDNA Erager (Takara). Real-time PCR was performed in an optical 96-well plate with an Applied Biosystems SteponeTM Real-Time PCR system. Each reaction contained 7.5 μl of 2 × SYBR Green Master Mix reagent, 0.5 μl of cDNA samples, and 0.6 μl of 10 μM gene-specific primers in a final volume of 15 μl. The thermal cycle used was as follows: 95°C for 10min; and 40 cycles of 95°C for 30 s, 55°C for 30 s, and 72°C for 30 s. All the primers used for quantitative RT-PCR are listed in Supplementary Table S1. The relative quantification method (Delta-Delta cycle threshold) was used to evaluate quantitative variation between the replicates examined. The PCR signals were normalized to those of actin or rice polyubiquitin (RubQ1).

### Analysis of the phloem sap

The protocols described by Corbesier *et al.* (2003) were used for collection of phloem sap. Briefly, newly formed leaves were detached at their petiole bases, and the petioles were recut in medium containing 20mM EDTA-K_2_ (pH 7.5). The leaves collected from each individual plant were placed in a 2ml microcentrifuge tube with their petioles immersed in 1.5ml of 15mM EDTA-K_2_ (pH 7.5). The tubes were placed in airtight transparent plastic containers in an illuminated growth room for 4h to dissolve the phloem sap in EDTA solution (Corbesier *et al.*, 2003); the atmosphere was water saturated (to prevent uptake of EDTA solution by the leaves). Iron concentration in the collected phloem sap was measured by Inductive Coupled Plasma Mass Spectrometry (ICP-MAS). The leaves were dried at 80°C and weighed.

### Statistical analysis

Analysis of variance was conducted between different treatments. The significant differences between treatments were evaluated by LSD multiple range tests (*P ≤* 0.05) using SAS statistical software.

## Results

### Exogenous application of EBR enhanced leaf symptoms of Fe deficiency

**Fig. 1.**
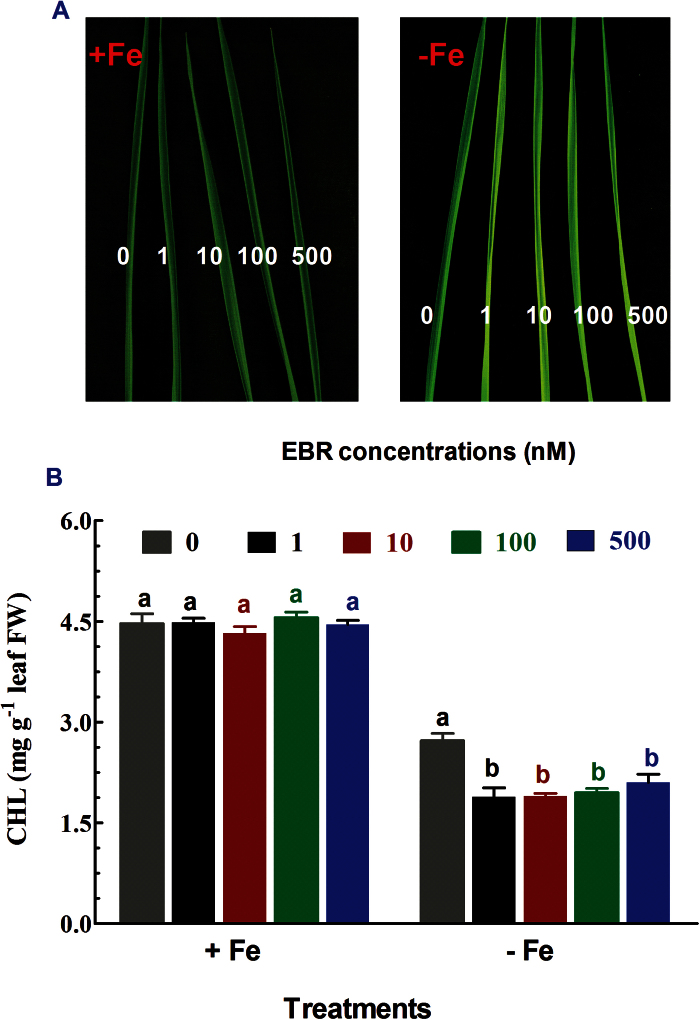
Effect of EBR on chlorophyll (CHL). (A) Photographs of 10-d-old WT rice seedlings exposed to Fe-sufficient or Fe-deficient medium with or without different concentrations of EBR (0, 1, 10, 100, and 500nM) for 2 weeks. (B) The chlorophyll concentrations in WT rice leaves were determined following treatment of WT seedlings of –Fe and +Fe plants with different concentrations of EBR (0, 1, 10, 100, and 500nM) for 2 weeks. Data are means ± SE (*n* = 4). Means with different letters are signiﬁcantly different (*P* ≤ 0.05) with regard to treatments.

To test whether BR is involved in Fe deficiency-induced changes in physiological processes in rice plants, the effect of exogenous EBR at varying concentrations (0–500nM) on cholorophyll concentrations of rice seedlings grown in Fe-sufficient and Fe-deficient media was studied. Apparent chlorosis was observed in young leaves of rice seedlings grown in Fe-deficient medium for 2 weeks (Fig. 1A), leading to a decrease in cholorophyll concentration (Fig. 1B). Furthermore, the chlorosis became more evident by application of EBR to Fe-deficient seedlings. In contrast, application of EBR had no apparent effect on leaf cholorosis in Fe-sufficient seedlings (Fig. 1A). Accordingly, treatments with EBR led to a signiﬁcant decrease in chlorophyll concentration of Fe-deﬁcient plants, while the same treatment had no effect on chlorophyll concentration in Fe-sufﬁcient seedlings (Fig. 1B). The reduction in chlorophyll concentration in Fe-deficient seedlings by EBR occurred at a concentration of 1nM, and no further reduction in the chlorophyll concentration was observed when EBR concentration was increased up to 500nM, suggesting that the effect of EBR on the chlorophyll concentration is independent of EBR dose, and that BR may be an important signal during Fe deficiency. Although 1nM EBR can have the negative effect, at >100nM it can partly restore the phenotype of the *d2-1* mutant. Therefore, an EBR concentration of 100nM was used throughout this study, which can partly restore growth of *d2-1* and has no effect on growth of WT plants. Since rice can take up both Fe^2+^- and Fe^3+^-MA, in addition to Fe-EDTA, we also investigated the effects of EBR on plants with different forms of Fe present in the medium (FeSO_4_, Fe^3+^EDTA, and FeCl_3_). Similar to FeEDTA, EBR-induced leaf chlorosis was also detected under conditions of deficiency in FeSO_4_, Fe^3+^EDTA, or FeCl_3_ in the growth medium (Supplementary Figure S1). These results indicate that the effect of BR on Fe nutrition is independent of Fe species in the growth medium. We therefore focused on the effect of BR on rice plants with FeEDTA in the medium throughout our study.

**Fig. 2.**
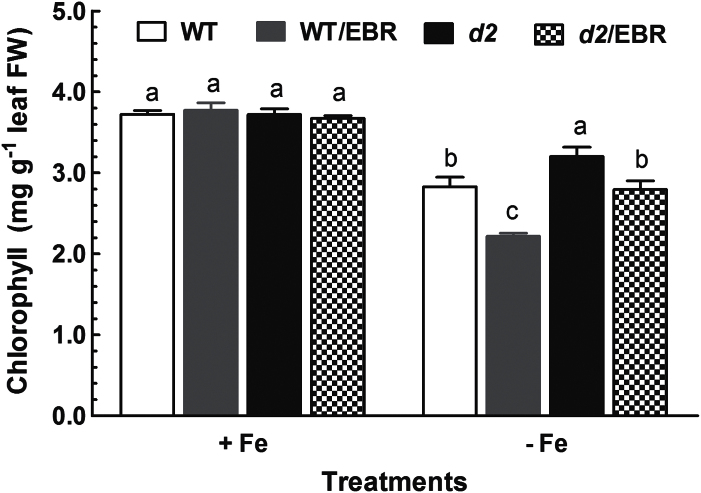
Effect of EBR on chlorophyll of the WT and *d2-1* mutant. Ten-d-old WT and *d2-1*mutant seedlings were exposed to Fe-sufficient and Fe-deficient medium with or without 100nM EBR for 2 weeks. After treatments, chlorophyll concentration in leaves were determined. Data are means ± SE (*n* = 4). Means with different letters are signiﬁcantly different with regard to treatments (*P* ≤ 0.05).

The involvement of BR in the mediation of Fe deficiency-induced changes in physiological processes was further evaluated using a BR-deficient mutant *d2-1*. Similar to WT plants, Fe deficiency also decreased leaf chlorophyll in the *d2-1* mutant, and exogenous application of EBR enhanced Fe-deficiency-induced leaf chlorosis in young leaves of *d2-1* seedlings (Fig. 2). However, the *d2-1* mutant had a higher chlorophyll concentration than the WT plant, and the magnitude of decrease in chlorophyll concentration by EBR was much greater in WT plants than in *d2-1* mutants under Fe- deficient conditions (Fig. 2), indicating that the WT is more sensitive to EBR than *d2-1* mutant plants under Fe-deficient conditions. In contrast, application of EBR had no apparent effect on foliar chlorophyll concentration in Fe-sufficient *d2-1* mutant seedlings (Fig. 2).

### BR-deficient mutant *d2-1* was less sensitive to Fe deficiency and EBR

**Fig. 3.**
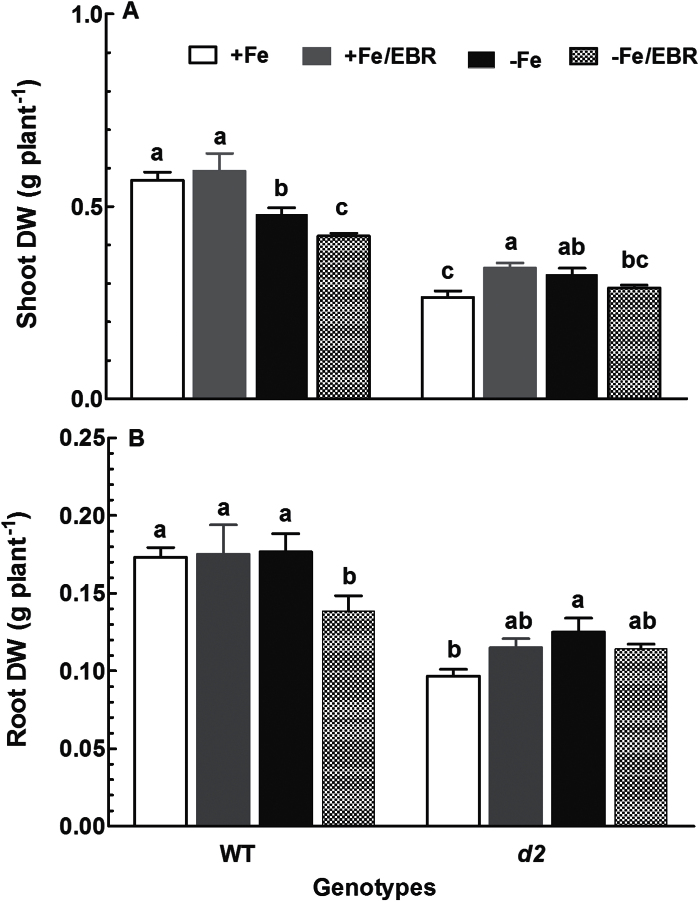
Effect of EBR on (A) shoot and (B) root dry weight (DW) of WT and *d2-1* plants. Ten-d-old WT and *d2-1* seedlings were exposed to Fe-sufficient and Fe-deficient medium with or without 100nM EBR for 2 weeks. After treatments, the dry biomass was measured. Data are means ± SE (*n* = 4). Means with different letters are signiﬁcantly different (*P* ≤ 0.05) within the same genotype.

In addition to chlorophyll, dry mass of shoots and roots of WT rice seedlings was significantly reduced when they were grown in Fe-deficient medium. A further reduction in dry weight of shoots and roots of Fe-deficient seedlings was found in the presence of EBR in the growth medium, while no effect of EBR on biomass of Fe-sufficient WT seedlings was observed (Fig. 3A, B). Both shoot and root biomass of *d2-1* mutant was significantly lower than that of WT plants when grown under Fe-sufficient conditions (Fig. 3A, B). In contrast to the WT, an increase in dry weight of the shoots and roots of the *d2-1* mutant was observed under Fe-deficient conditions (Fig. 3B). There was a significant increase in shoot dry weight and a slight increase in root dry weight in the *d2-1* mutant by EBR application under Fe-deficient and Fe-sufficient conditions, respectively (Fig. 3A, B).

**Fig. 4.**
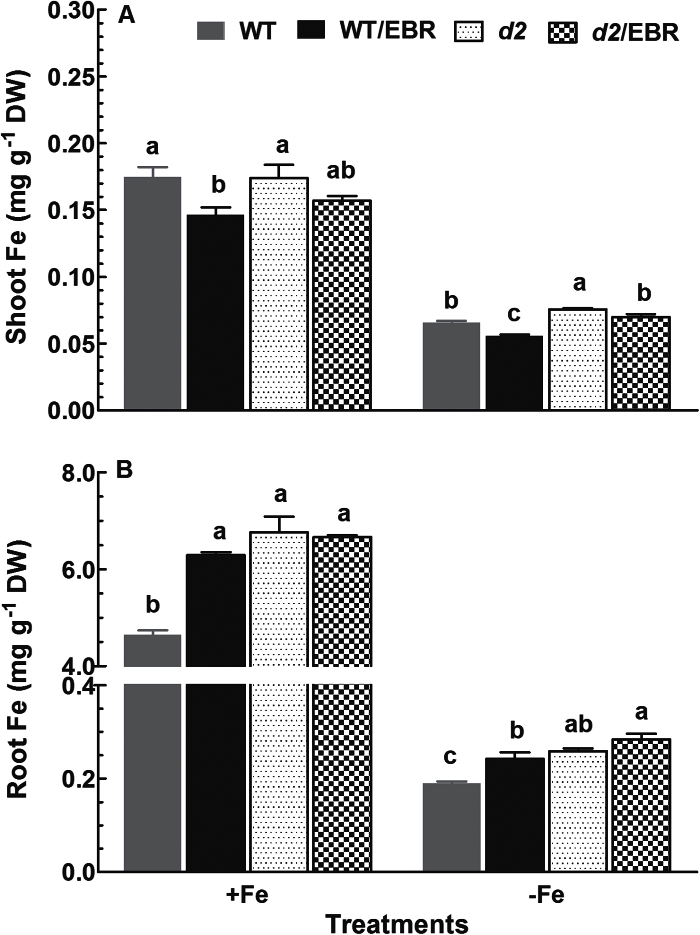
Effect of EBR on Fe concentrations in (A) shoots and (B) roots of wild-type and *d2-1* mutant plants. Ten-d-old WT seedlings were exposed to Fe-sufficient and Fe-deficient medium with or without 100nM EBR for 2 weeks. After treatments, Fe concentration was measured. Data are means ± SE (*n* = 4). Means with different letters are signiﬁcantly different (*P* ≤ 0.05) within the same Fe treatment.

Exposure of rice seedlings to Fe-deficient medium led to a significant decrease of Fe concentrations in roots and shoots in both the WT and the *d2-1* mutant (Fig. 4). Also, Fe concentrations in roots of the *d2-1* mutant were signiﬁcantly higher than those of WT plants under both Fe-deficient and Fe-sufficient conditions (Fig. 4B). In contrast to Fe concentrations in roots, concentrations in shoots of *d2-1* plants were higher than those of the WT grown in Fe-deficient medium (Fig. 4A), but no differences in shoot Fe concentrations among WT and *d2-1* under Fe-sufficient conditions were observed (Fig. 4A). Exogenous application of EBR had contrasting effects on Fe concentrations in roots and shoots of WT rice seedlings regardless of Fe supply, such that treatment with EBR led to decreases in Fe concentrations in shoots by 16 and 15% in Fe-sufﬁcient and Fe-deﬁcient seedlings, respectively (Fig. 4A), while there were 35 and 27% increases in root Fe concentrations of both Fe-sufﬁcient and Fe-deﬁcient seedlings when treated with EBR (Fig. 4B). In contrast to WT plants, EBR had no effect on Fe concentrations of *d2-1* in shoots and roots under both Fe-sufficient and Fe-deficient conditions (Fig. 4).

To test whether the EBR-induced changes in Fe concentrations in shoots and roots were specific to Fe, the effect of EBR on other metals concentrations were studied under Fe-sufficient conditions. In contrast to Fe, Mg and Zn concentrations in shoots and roots were not affected by EBR (Supplementary Figure S2). The concentration of Mn in roots was not affected by EBR, but it was significantly increased by EBR in shoots (Supplementary Figure S2). These results suggest that BR has a specific effect on Fe nutrition in shoots and roots of rice.

### Expression patterns of genes related to Fe deficiency in roots

**Fig. 5.**
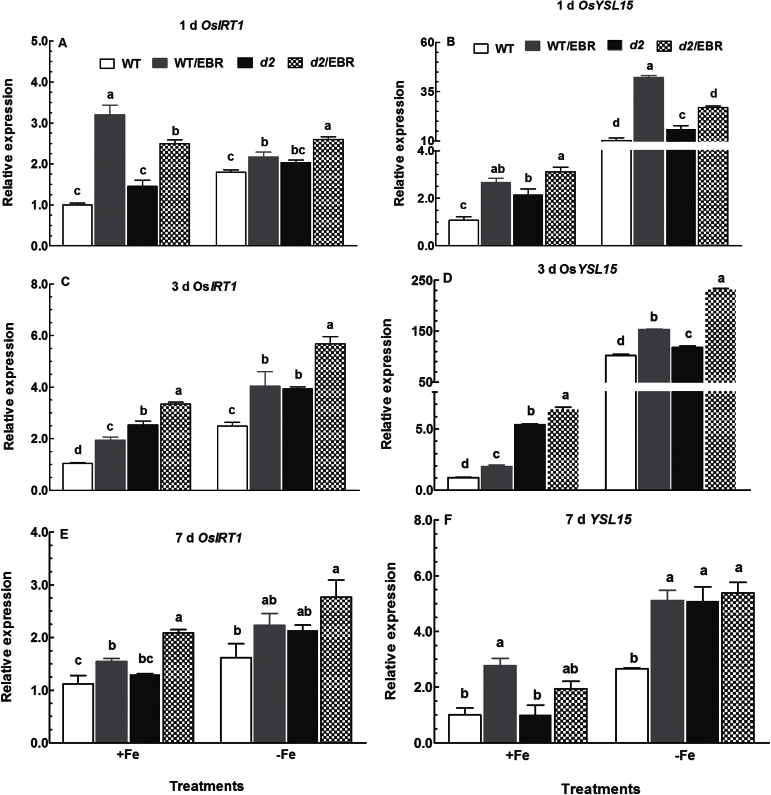
Time-course quantitative RT-PCR analysis of Fe uptake-related genes in roots: effect of EBR on Os*IRT1* and Os*YSL15* expression in roots of WT and *d2-1* plants under +Fe and –Fe conditions. Data are means ± SE of three biological replicates. Means with different letters are signiﬁcantly different within the same gene (*P* ≤ 0.05).

OsIRT1 and OsYSL15 are directly involved in Fe uptake from the rhizosphere (Conte and Walker, 2011). Therefore, we examined the responses of these genes to EBR in WT and *d2* mutants under both Fe-deficient and Fe-sufficient conditions. Upregulation of Os*IRT1* and Os*YSL15* was observed after 1-d treatment with Fe deficiency in both the WT and *d2-1* mutants (Fig. 5A, B). The expression of Os*IRT1* and Os*YSL15* was significantly upregulated by EBR under Fe-sufficient conditions (Fig. 5A, B), and EBR further enhanced the Fe deficiency-induced expression of Os*YSL15* and Os*IRT1* in WT and *d2-1* plants (Fig. 5A, B). Moreover, longer exposure (3 and 7 d) of WT and *d2-1* seedlings to Fe-deficient medium and EBR also led to upregulation of Os*IRT1* and Os*YSL15* (Fig. 5C–F). However, the magnitude of upregulation of Os*IRT1* and Os*YSL15* by EBR was much greater in WT plants than in *d2-1* mutants under both Fe-sufficient and Fe-deficient conditions. In general, similar effects of EBR and Fe deficiency on expression of Os*IRT1* and Os*YSL15* were observed after treatments with Fe deficiency and EBR for 3 and 7 d (Fig. 5C–F), suggesting that both short- and long-term treatment with Fe deficiency and EBR can alter expression patterns of Os*IRT1* and Os*YSL15*. The expression patterns of Os*IRT1* and Os*YSL15* in WT and *d2-1* mutant plants grown in Fe-sufficient and Fe-deficient media were also compared. Expression levels of Os*IRT1* and Os*YSL5* in *d2-1* mutants were higher than in WT plants grown in Fe-sufficient medium after treatment for 1 and 3 d, and thereafter the mutant and WT plants had comparable expression levels of Os*YSL15* (Fig. 5A–F). Exposure of WT and *d2-1* mutant plants to Fe-deficient medium led to similar changes in expression patterns of Os*IRT1* (Fig. 5 A–F). In contrast to Os*IRT1* expression, the Fe deficiency-induced upregulation of Os*YSL15* expression in *d2-1* mutant plants was less than in WT plants after exposure for 1 and 3 d.

**Fig. 6.**
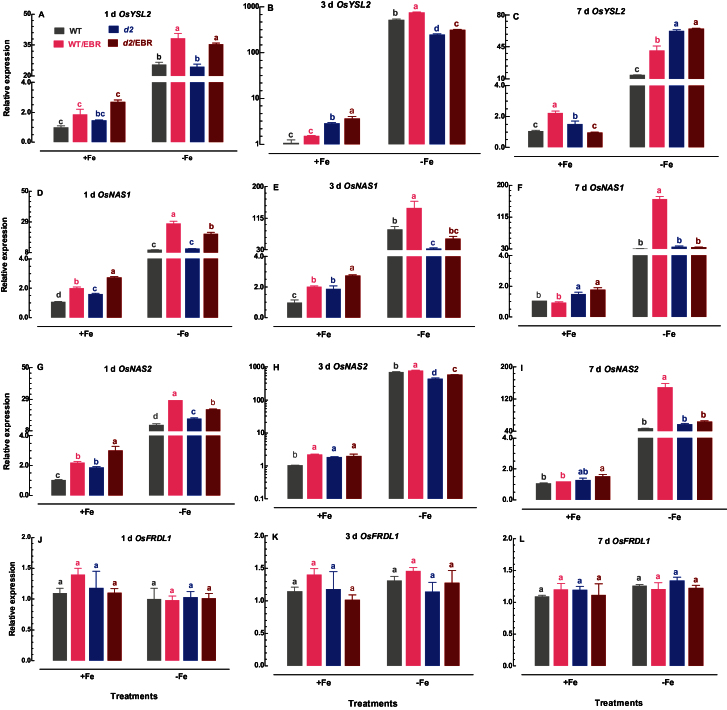
Time-course quantitative RT-PCR analysis of Fe translocation related genes in roots: effect of EBR on Os*YSL2*, Os*NAS1*, Os*NAS2*, and Os*FRDL1* expression in roots of WT and *d2-1* plants under +Fe and –Fe conditions. Data are means ± SE of three biological replicates. Means with different letters are signiﬁcantly different within the same Fe treatment (*P* ≤ 0.05). (This figure is available in colour at *JXB* online)

YSL2 and NA have been proposed to mediate long-distance translocation of Fe, and their expression is upregulated under Fe-deficient conditions (Inoue *et al.*, 2003; Ishimaru *et al.*, 2010). Os*FRDL1* encodes a citrate transporter that is localized at the pericycle cells, and is essential for translocation of Fe-citrate complex to the shoot (Yokosho *et al.*, 2009). There were increases in expression levels of Os*YSL2*, Os*NAS1*, and Os*NAS2* in Fe-sufficient WT and *d2-1* plants upon addition of EBR to the medium (Fig. 6A–I). In addition to EBR, exposure of WT and *d2-1* plants to Fe-deficient medium also led to similar increases in expression levels of Os*YSL2*, Os*NAS1*, and Os*NAS2* in terms of magnitude and time course (Fig. 6A–I). In addition, the magnitude of upregulation of Os*YSL2*, Os*NAS1* and Os*NAS2* expression was much greater in WT and *d2-1* mutants challenged by Fe deficiency and EBR together than by treatment with Fe deficiency and EBR alone (Fig. 6A–I), suggesting that Fe deficiency and EBR may have additive effects on the expression of these genes. In contrast to Os*YSL2*, Os*NAS1*, and Os*NAS2*, the expression level of Os*FRDL1* was not responsive to treatments with Fe deficiency and EBR alone or Fe deficiency and EBR together (Fig. 6J–L). Transcript levels of Os*NAS1* and Os*NAS2* in *d2-1* plants were generally higher than in the WT under Fe-sufficient conditions (Fig. 6A–I). Treatment with Fe deficiency also induced upregulation of expression of Os*YSL2*, Os*NAS1*, and Os*NAS2* in *d2-1* mutant plants (Fig. 6A–I). However, the magnitude of the upregulation in *d2-1* plants was less than the Fe deficiency-induced upregulation of these genes in WT plants (Fig. 6A–I). No apparent differences in expression levels of Os*FRDL1* between WT and *d2-1* plants under both Fe-sufficient and Fe-deficient conditions were observed (Fig. 6J–L).

### Expression patterns of transcription factor Os*IRO2* in roots

**Fig. 7.**
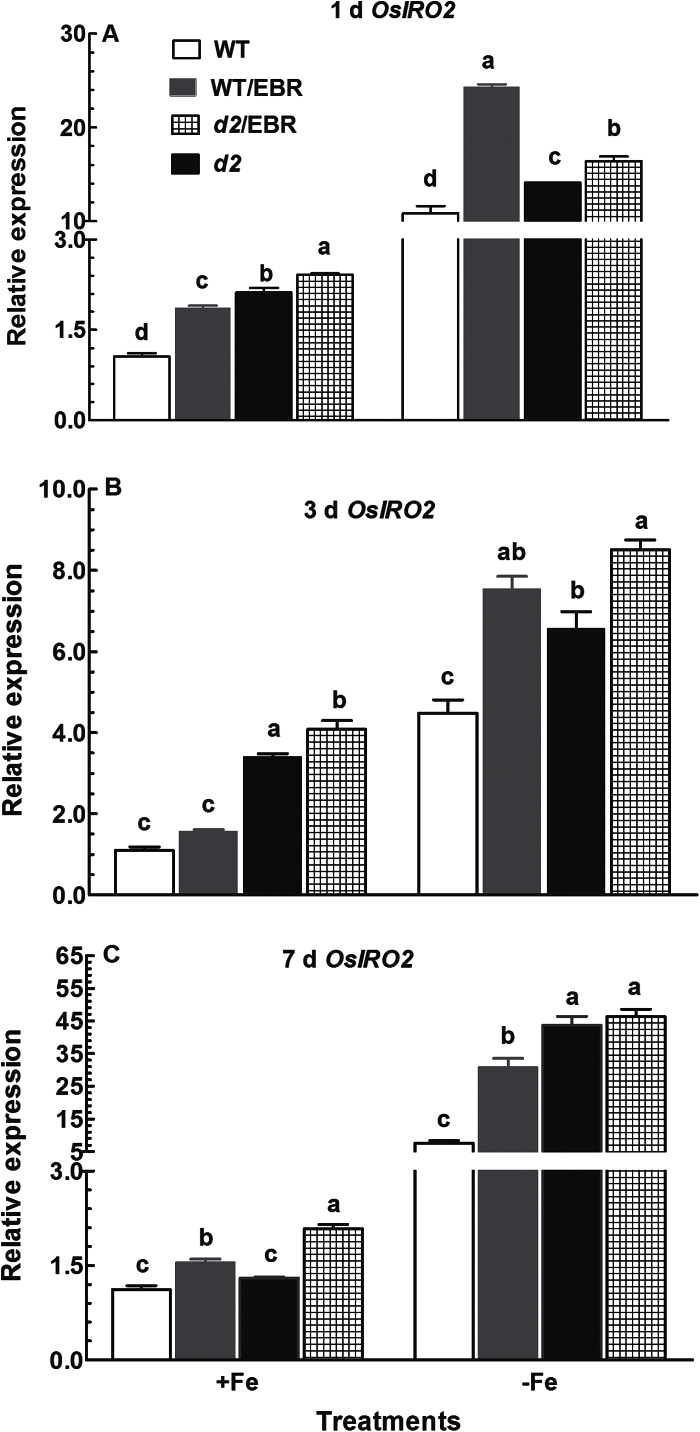
Time-course quantitative RT-PCR analysis of Os*IRO2* in roots: effect of EBR on Os*IRO2* expression in roots of WT and *d2-1* plants under +Fe and –Fe conditions. Data are means ± SE of three biological replicates. Means with different letters are signiﬁcantly different within the same Fe treatments (*P* ≤ 0.05).

The rice bHLH protein OsIRO2 is an essential regulator involved in mediation of Fe uptake (Ogo *et al.*, 2007). The expression level of Os*IRO2* was enhanced after addition of EBR to Fe-sufficient medium in both WT and *d2-1* plants (Fig. 7A–C). In addition to EBR, expression of Os*IRO2* was also upregulated by exposure to Fe-deficient medium, and the effect of EBR and Fe deficiency on expression of Os*IRO2* was additive, such that the expression levels were highest in plants exposed to Fe-deficient medium containing EBR (Fig. 7A–C). However, the magnitude of the upregulation by EBR in *d2-1* plants was less than in WT plants under both Fe-sufficient and Fe-deficient conditions. The abundance of Os*IRO2* transcript was higher in *d2-1* plants than in WT plants under Fe-sufficient conditions after 1 and 3 d of treatment, and became comparable after 7-d exposure to Fe-deficient medium (Fig. 7A–C).

### Expression patterns of Os*YSL2*, Os*NAS1*, and Os*NAS2* in shoots

**Fig. 8.**
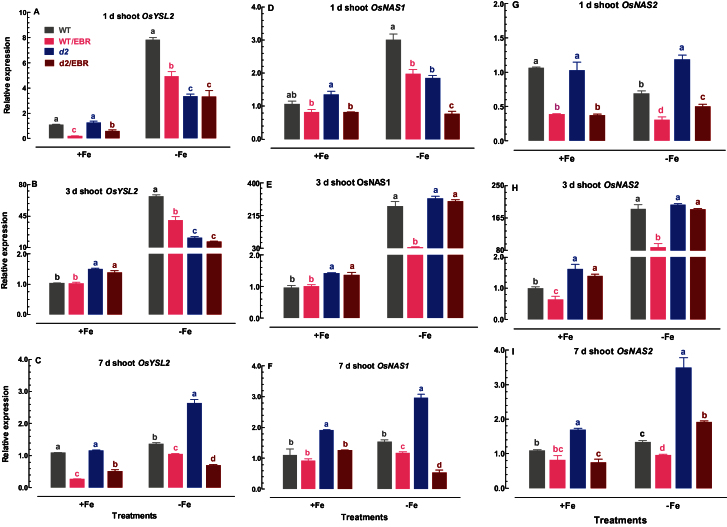
Time-course quantitative RT-PCR analysis of Os*YSL2*, Os*NAS1*, and Os*NAS2* in shoots: Effect of EBR on Os*YSL2* expression in shoots of WT and *d2-1* plants under +Fe and –Fe conditions. Data are means ± SE of three biological replicates. Means with different letters are signiﬁcantly different within the same Fe treatment (*P* ≤ 0.05). (This figure is available in colour at *JXB* online)

OsYSL2 is a critical Fe-nicotianamine transporter involved in the translocation of Fe, especially in shoots and endosperms (Ishimaru *et al.*, 2010). The responsiveness of Os*YSL2* in shoots of WT and *d2-1* mutants to EBR under Fe-sufficient and Fe-deficient conditions was monitored. The expression level of Os*YSL2* was suppressed by addition of EBR to the Fe-sufficient medium for 1 and 7 d in both WT and *d2-1* plants (Fig. 8A–C). In contrast, exposure of WT and *d2-1* seedlings to Fe-deficient medium led to a sustained upregulation of Os*YSL2* (Fig. 8A–C). Treatment of Fe-deficient seedlings with EBR led to a suppression of Os*YSL2* expression at all experimental stages, such that the expression level was lower in plants exposed to Fe-deficient medium with EBR than in plants exposed to Fe-deficient medium without EBR (Fig. 8A–C). The expression level of Os*YSL2* in *d2-1* plants was slightly higher than in the WT under Fe-sufficient conditions (Fig. 8A–C).

It has been demonstrated that NA is essential for Fe mobilization from the vasculature into the interveinal tissues, and that loss of NA leads to leaf chlorosis (Morrissey and Guerinot, 2009). In the present study, we tested the expression patterns of Os*NAS1* and Os*NAS2* in shoots under varying conditions. The expression levels Os*NAS1* was not affected by EBR from 1 to 7 d in WT plants, but the expression level of Os*NAS2* was suppressed by addition of EBR to Fe-sufficient medium for 1 and 3 d in WT plants (Fig. 8D–I). Treatments of EBR inhibited both Os*NAS1* and Os*NAS2* expression in *d2-1* plants at 1 and 7d under Fe-sufficient conditions (Fig. 8D–I). Similar to Os*YSL2*, treatment of Fe-deficient seedlings of WT plants with EBR led to suppression of Os*NAS1* and Os*NAS2* at all experimental stages (Fig. 8D–I). The expression level of Os*NAS1* and Os*NAS2* was depressed by EBR in *d2-1* mutants at 1 and 7 d under Fe-deficient conditions (Fig. 8D–I). The expression level of Os*NNAS1* and OsNAS2 in *d2-1* plants was slightly higher than in WT under Fe-sufficient conditions (Fig. 8D–I).

### Fe concentrations in the phloem sap

**Fig. 9.**
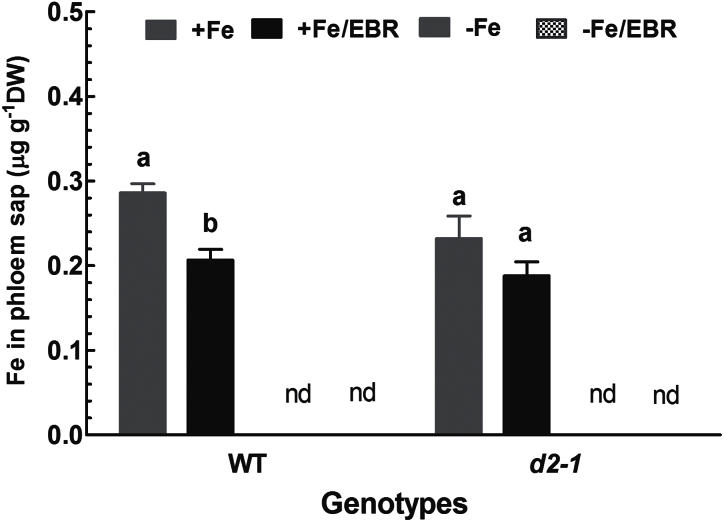
Effect of EBR on Fe concentration in phloem sap of the WT and *d2-1* mutant. Ten-d-old WT and d2-1 seedlings were exposed to Fe-sufficient and Fe -deficient medium with or without 100nM EBR for 2 weeks. After treatments, phloem sap was collected and Fe concentration was measured. Data are means ± SE (*n* = 4). Means with different letters are signiﬁcantly different within the same genotype (*P* ≤ 0.05).

OsYSL2 is a metal-NA transporter responsible for translocation of Fe in the phloem of rice (Koike *et al.*, 2004). To test whether the EBR-induced inhibition of Os*YLS2* expression can affect transport or translocation of Fe in the phloem, Fe concentrations in the phloem sap were measured. Fe concentrations in the phloem of the WT were significantly reduced in the presence of EBR under Fe-sufficient conditions (Fig. 9). In contrast, Fe concentrations in the phloem of Fe-sufficient *d2-1* plants were not changed by EBR. Little Fe could be detected in the phloem sap when rice plants were grown in Fe-deficient medium in the absence and presence of EBR (Fig. 9). These results indicate that EBR inhibits the translocation of Fe via the phloem.

### Expression patterns of genes related to BR biosynthesis and signal transduction

**Fig. 10.**
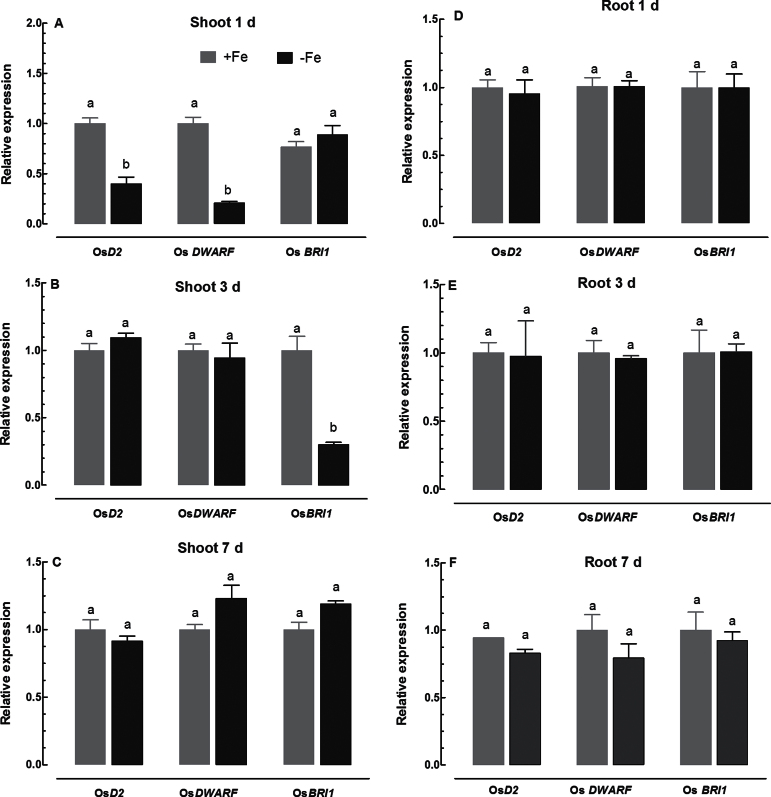
Time-course quantitative RT-PCR analysis of genes related to BR biosynthesis and signal transduction in shoots and roots: effect of Fe deficiency on Os*D2,* Os*DWARF*, and Os*BRI1* expression in shoots (A, B, and C) and roots (D, E, and F) of WT plants. Data are means ± s.e. of three biological replicates. Means with different letters are signiﬁcantly different within the same gene (*P* ≤ 0.05)

To dissect the network associated with BR-dependent Fe uptake and translocation, the response of BR biosynthesis and signalling to Fe deficiency was examined at the transcriptional level in shoots and roots. The expression levels of *WRARF* and *D2*, which encode proteins involved in BR biosynthesis (Hong *et al.*, 2002; Hong *et al.*, 2003), and *BRI1*, which encodes a BR receptor (Morinaka *et al.*, 2006), were substantially suppressed after exposure to Fe-deficient medium for 1 d (Fig. 10A–C). The expression levels of *WRARF* and *D2* in Fe-deficient and Fe-sufficient seedlings became comparable after exposure to Fe-deficient medium for 3 and 7 d (Fig. 10B, C). The Fe deficiency-induced suppression of *BRI1* was also observed after 3 d of exposure to Fe-deficient medium, but the expression level became comparable to that in Fe-sufficient plants after exposure to Fe-deficient medium for 7 d (Fig. 10B, C). In contrast, expression of the three genes in roots was relatively constant after exposure to Fe-deficient medium up to 7 d (Fig. 10D–F). These results suggest that both BR biosynthesis and signal transduction in shoots may be closely regulated by Fe status in plants.

## Discussion

There is increasing evidence demonstrating the involvement of BR in the mediation of abiotic stress responses (Sasse, 2003). However, few studies have investigated the role of BR in the response of plants to deficiency in mineral nutrients. Our previous studies revealed that BR is involved in the response of cucumber, a strategy I plant, to Fe deﬁciency by negatively regulating Cs*FRO1*, Cs*IRT1*, and Fe translocation from roots to shoots (Wang *et al.*, 2012). Since the mechanisms by which Fe uptake and translocation differ between strategy I and II plants, different mechanisms may account for the effect of BR on their Fe acquisition. In the present study, we demonstrate that BR may also play a role in the regulation of the response of rice plants, a strategy II plant, to Fe deficiency using wild-type and BR-deficient mutant *d2-1*. We further show that an exogenous application of EBR rendered WT plants more sensitive to Fe deficiency by interfering with Fe homeostasis, and that the effect is specific to Fe. Rice mutant *d2-1*, which is deficient in endogenous BR levels due to disruption of BR biosynthesis, exhibited enhanced tolerance to Fe-deficiency, and the increased tolerance could be partly reversed by EBR complementation. These results suggest that BR is involved in the response of strategy II plants to Fe deficiency. Two mutant lines, *d2-1* and *d2-2*, were originally generated by Hong *et al.* (2003). The *d2-1* mutant exhibits more severe phenotypes than the *d2-2* mutant, and the two types of plant are allelic (Hong *et al.*, 2003). Therefore, the results obtained from the *d2-1* mutant would not undermine our conclusion.

Exogenous application of EBR to WT rice seedlings enhanced Fe deficiency-induced leaf chlorosis (Fig. 1), but the BR-deficient mutant *d2-1* was more tolerant to Fe deficiency than WT plants (Fig. 2). The growth rate was lower in the *d2-1* mutant than in WT plants, which may lead to a lower nutrient demand in the *d2-1* mutant. To test whether the enhanced tolerance of *d2-1* plants to Fe deficiency is not caused by their lower growth rate, we measured other mineral nutrients and compared the response of *d2-1* and *d61-2* to Fe deficiency. The *d61-2* mutation was caused by loss of function of OsBRI1, a putative BR receptor (Hong *et al.*, 2003), and *d61-2* mutants exhibit reduced growth compared to WT plants under normal growth conditions (Morinaka *et al.*, 2006). In contrast to increased Fe concentrations in *d2-1*, there were no differences in Mg and K concentrations in shoots and roots between WT and *d2-1* under Fe-sufficient conditions (data not shown). Our data also showed that *d2-1* and *d61-2* exhibited similar shoot dry weight under Fe-deficient conditions, but *d61-2* was more sensitive to Fe deficiency than *d2-1* in terms of chlorophyll concentration, shoot dry weight, and shoot Fe concentrations (Supplementary Figure S3). These results suggest that the enhanced tolerance to Fe deficiency of the *d2-1* mutant may not simply be accounted for by its reduced growth rate, and that BR may be specifically involved in Fe uptake, transport, and translocation processes in rice. OsIRT1 and OsYSL15 are directly involved in Fe uptake from the rhizosphere in rice (Conte and Walker, 2011). Our results showing that Fe deficiency significantly enhanced expression of Os*IRT1* and Os*YSL15* (Fig. 5) are in agreement with those reported in the literature. Expression of Os*IRT1* and Os*YSL15* in the *d2-1* mutant was higher than in WT plants under Fe-sufficient conditions (Fig. 5), which may underpin the higher Fe accumulation in roots of the *d2-1* mutant than in roots of WT plants. After uptake, Fe is transported in the complexed forms once it is loaded into the xylem (Conte and Walker, 2011). OsFRDL1, a citrate transporter localized at the rice root pericycle cells, is necessary for efficient translocation of Fe-citrate complex to shoots (Yokosho *et al.*, 2009). Expression of Os*FRDL1* was relatively insensitive to Fe deficiency and EBR (Fig. 6). A similar insensitivity of Os*FRDL1* to Fe deficiency has been reported in the literature (Yokosho *et al.*, 2009). Although citrate plays an important role in long-distance transport of Fe in rice, knockout of Os*FRDL1* results in mild defects in Fe homeostasis compared with the severe phenotype of the *Arabidopsis frd3* mutant (Kobayashi and Nishizawa, 2012), suggesting that alternative chelators may exist in rice to mediate transport of Fe in the xylem. In addition to citrate, NA levels have a significant effect on metal homeostasis (Morrissey and Guerinot, 2009). Overexpression of NAS in tobacco and *Arabidopsis* increases NA levels in shoots, resulting in increased Fe in the shoots (Takahashi *et al.*, 2003; Kim *et al.*, 2005). However, it is unclear whether these changes are the result of greater root to shoot translocation facilitated by NA or increased metal uptake in the roots driven by the creation of new Fe sinks in the shoot (Morrissey and Guerinot, 2009). In rice, YSL2 and NA have been proposed to mediate long-distance translocation of Fe and/or are responsible for the phloem transport of Fe in shoots (Inoue *et al.*, 2003; Koike *et al.*, 2004; Ishimaru *et al.*, 2010). In our study, we found that expression of Os*YSL2*, Os*NAS1*, and Os*NAS2* in shoots was significantly depressed by EBR treatment (Fig. 8), and that Fe concentrations in phloem sap of WT plants were also decreased by EBR application (Fig. 9). EBR treatment may decrease shoot NA and inhibit Fe translocation from roots to shoots or decrease the Fe sink in shoots. The suppression of shoot Os*YSL2* may suppress the unloading of Fe from the phloem to mesophyll cells and lead to a low Fe concentration in protoplasts of leaf mesophyll cells and enhanced leaf chlorosis. The lowered shoot Fe by BR feedbacks to roots enhanced the expression of many genes involved in Fe homeostasis, leading to Fe accumulation in roots, but decreased biomass and Fe of shoots.

Os*IRO2* is an essential transcription factor modulating Fe uptake and translocation in rice plants (Kobayashi and Nishizawa, 2012). Expression of Os*IRO2* is often upregulated upon exposure to Fe-deficient medium, thus activating the expression of genes responsible for Fe homeostasis in roots (Ogo *et al.*, 2007, 2011). Our results are in agreement with those reported in the literature. Moreover, our results showed that EBR significantly enhanced Os*IRO2* expression in both Fe-sufficient and Fe-deficient WT rice plants (Fig. 7), which may account for the EBR-induced upregulation of Fe homeostasis-related genes, such as Os*YSL15*, Os*NAS1*, and Os*NAS2* (Fig. 6). Similar to EBR application, a higher expression of Os*IRO2* and genes related to Fe homeostasis was observed by BR deficiency in the *d2-1* mutant (Fig. 6). Root Fe and expression levels of genes related to Fe homeostasis in the WT and *d2-1* mutant were increased or upregulated by exogenous EBR application and endogenous BR defects, respectively, but the mechanisms may be different. Expression of Os*IRT1* and Os*YSL15* in *d2-1* mutants was higher in *d2-1* than in WT plants under Fe-sufficient conditions (Fig. 5), which may account for the higher Fe uptake and accumulation in roots of *d2-1* mutants than in roots of WT plants. In addition to efficient Fe-uptake systems of roots in *d2-1*, the expression of genes related to Fe transport and translocation in shoots of *d2-1* was also higher than that in WT plants (Fig. 8). Thus, BR-deficient mutant *d2-1* had comparable shoot Fe concentrations with WT plants under Fe-sufficient conditions (Fig. 4A), suggesting that, for the *d2-1* mutant, upregulation of genes associated with Fe homeostasis may account for the higher Fe concentrations in shoots. These may provide an explanation for the greater tolerance of *d2-1* to Fe deficiency than WT plants. The efficient uptake and translocation of Fe may make the *d2-1* mutant more tolerant to Fe deficiency and less responsive to EBR treatment, as evidenced by less suppression of plant biomass and Fe concentrations in the *d2-1* mutant than the WT when grown in Fe-deficient medium, and lower expression levels of genes associated with Fe uptake and translocation at early stages of Fe deficiency. Unlike the *d2-1* mutant, exogenous EBR suppressed the expression of genes related to Fe transport and translocation in shoots (Fig. 8), leading to reductions in shoot Fe concentrations of WT plants (Fig. 4). Decreased shoot Fe concentration by EBR enhanced leaf chlorosis, which may feed back to the root and activate the expression of Fe homeostasis-related genes, thus leading to Fe accumulation in roots. The symptoms in rice seedlings treated with exogenous BR are comparable to those in *Arabidopsis* mutant *frd3*, including leaf chlorosis, constitutive expression of genes associated with Fe uptake, and low Fe level in the plastid, even under Fe-sufficient conditions (Rogers and Guerinot, 2002).

**Fig. 11.**
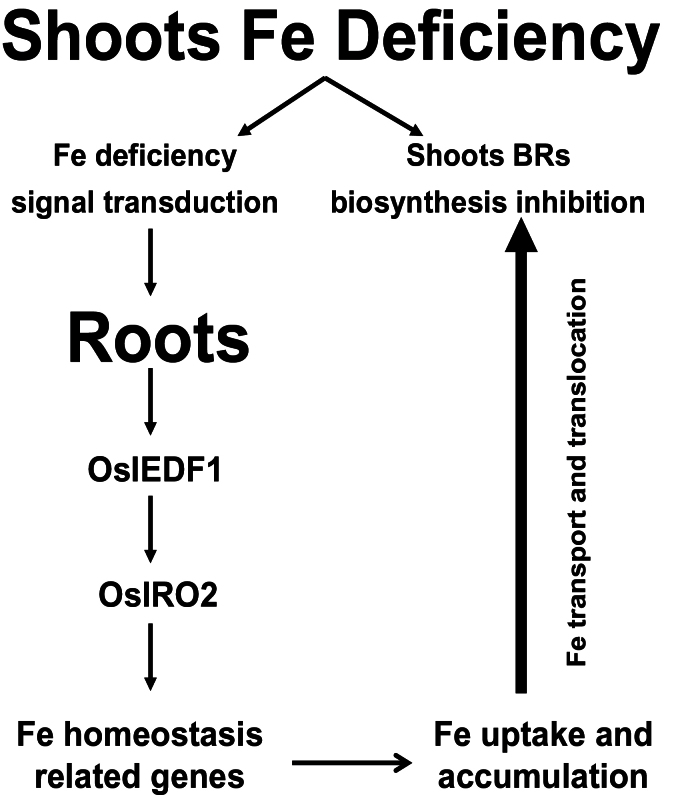
A putative working model to illustrate the possible role of BR in regulating the response of rice plants to Fe deficiency. Pathways possibly responsible for Fe uptake or transport and translocation from roots to shoots are separated. Shoot Fe deficiency could induce signal transduction from shoots to roots, and activate Fe mobilization and uptake by roots. On the other hand, Fe deficiency could decrease BR levels in shoots and inhibit Fe transport and translocation from roots to shoots.

Exogenous application of BRs has been shown to improve stress tolerance by activating the BR signal transduction pathway and BR-regulated expression of stress-related genes (Kagale *et al.*, 2007). In the present study, we found that Fe concentrations in shoots were negatively regulated by BRs, but the signal transduction pathways remain to be dissected. In rice, Os*D2* and Os*DWARF* are responsible for biosynthesis of BRs, and Os*BRI1* is a receptor for BR (Yamamuro *et al.*, 2000; Hong et al., 2002, 2003). Under salt and drought stress, expression of salt- and drought-responsive genes is rapidly altered by BR (Kagale *et al.*, 2007). Also, expression of the BR receptor gene in rice can be modified within several hours by treatment with IAA (Sakamoto *et al.*, 2013). These indicate that plants can be quickly responsive to environmental and hormonal cues by modulating BR signalling cascades. Expression of Os*D2*, Os*DWARF*, and Os*BRI1* in roots of WT plants was not responsive to Fe deficiency (Fig. 10D–F). In contrast, expression of Os*D2* and Os*DWARF* in shoots was markedly suppressed by exposure to Fe-deficient medium for 1 d, while the Fe deficiency-induced inhibition of Os*BRI1* expression occurred after 3-d exposure to Fe-deficient medium (cf. Fig. 10). These results imply that BRs may be involved in regulation of Fe transport and translocation from roots to shoots, but the underlying mechanisms remain to be elucidated. In rice, the regulatory network involving Os*IDEF1*, Os*IDEF2*, and Os*IRO2* in response to Fe deficiency has been elucidated (Kobayashi *et al.*, 2007; Ogo *et al.*, 2008; Kobayashi *et al.*, 2009, 2012; Kobayashi and Nishizawa, 2012). Based on the information and our results, a putative working model is proposed to illustrate the possible role of BR in regulating the response of rice plants to Fe deficiency (Fig. 11). The iron deficiency signal transmits from shoot to root, and activates transcription factors Os*IDEF1* and Os*IRO2*, which in turn modulates the downstream targets involved in Fe homeostasis at the transcriptional level, leading to Fe uptake and accumulation in roots. In contrast, Fe deficiency inhibited BR biosynthesis, and the reduced endogenous BRs may facilitate Fe transport and translocation from roots to shoots. The exogenous application of EBR to rice seedlings would suppress the transport and translocation of Fe from roots to shoots, thus leading to a more severe phenotype of Fe deficiency in shoots and strengthen Fe deficiency signal. The enhanced signal of Fe deficiency will further upregulate downstream genes involved in Fe homeostasis, and lead to Fe uptake and accumulation in roots. BRs negatively regulated Fe transport and translocation from root to shoot in rice seedlings, but the mechanisms are not clear. Therefore, future research focusing on the interaction of BR with Fe transport and translocation in strategy II plants is warranted.

In conclusion, our results highlight the importance of BR as a signalling molecule involved in mediating the response of rice plants to Fe deficiency. More specifically, we demonstrate that BR plays a negatively regulatory role in control of transport and translocation from roots to shoots, thus indirectly modulating Fe mobilization and acquisition by possibly regulating Os*IRO2*.

## Supplementary data

Supplementary data can be found at *JXB* online.

Supplementary Table S1. Primers used for quantitative RT-PCR.

Supplementary Figure S1. Effect of EBR on chlorophyll concentrations in WT rice seedlings in the presence and absence of different forms of Fe (Fe^3+^, Fe^2+^).

Supplementary Figure S2. Effect of EBR on Mg, Mn, and Zn concentrations in shoots and roots of WT rice seedlings.

Supplementary Figure S3. Effect of Fe deficiency on chlorophyll concentrations, shoot and root dry weight, and Fe concentrations in shoots of *d2-1* and *d61-2* mutants.

Supplementary Figure S4. Quantitative RT-PCR analysis of OsD2 in roots and shoots of WT and *d2-1* mutant plants under different Fe supply conditions.

## Funding

This work was supported by the Natural Science Foundation of China (31101594) and State Key Laboratory of Vegetation and Environmental Change.

## Supplementary Material

Supplementary Data
