# Supplementary material for: Brassinosteroids are involved in Fe homeostasis in rice (Oryza sativa L.)
Source: J Exp Bot. 2015 Mar 14;66(9):2749–61. doi: 10.1093/jxb/erv079 (PMC4986876; doi:10.1093/jxb/erv079)
Supplement: Supplementary Data [file supp_66_9_2749__index.html]

Brassinosteroids are involved in Fe homeostasis in rice (Oryza sativa L.) — Brassinosteroids are involved in Fe homeostasis in rice (Oryza sativa L.) — Supplementary Data 

# Brassinosteroids are involved in Fe homeostasis in rice (*Oryza sativa* L.)

## Supplementary Data

Data files

**Files in this Data Supplement:**

- Supplementary Data - Supplementary Data
