# Supplementary material for: Brassinosteroids are involved in Fe homeostasis in rice (Oryza sativa L.)
Source: J Exp Bot. 2015 Mar 14;66(9):2749–61. doi: 10.1093/jxb/erv079 (PMC4986876; doi:10.1093/jxb/erv079)
Supplement: Supplementary Data [file supp_erv079_jexbot139881_file001.pdf]

Supplementary Table S1. The primers that were used for quantitative RT-PCR in the study.

Gene	Forward primer	Reverse primer
OsFRDL1	5'-TCACCAATGCTAAGGCCTGC-3'	5'-AACCACGGAAAACACCCTG-3'
OsYSL2	5'-TACAAAAGGGTAAGTGGTATCC-3'	5'-AAATG TATCCTCATCTGTCGC-3'
OsYSL15	5'-AACATAAGGGGGACTGGTAC-3'	5'-TGATTACCGCAATGATGCTTAG-3'
OsIRT1	5'-CGTC TTCTTCTTCTCCACCACGAC-3'	5'-GCAGCTGATGATCGAGTCTG ACC-3'
OsNAS1	5'-GTCTAACAGCCGGACGATCGAAAGG-3'	5'-TTTCTCACTGTCATACACAGATGGC-3'
OsNAS2	5′-TGAGTGCGTGCA TAGTAATCCTGGC-3′	5′-CAGACGGTCACAAACACCTCTTGC-3′
OsIRO2	5'-CTCCCATCGTTTCGGCTACCT-3'	5'-GCTGGGCACTCCTCGTTGATC-3'
OsBRI1	5' CAGCTACTTGGCTATCTTGAAGCTCAGC-3'	5'-CCATTCTTGTTGA AGGTGTACTCCGTGC-3'
OsD2/CYP90D2	5'-TTCAACCCATGGAGGTGGAA-3'	5'-GCACGGTGGGGAAGTTGACGA-3'
OsDWARF	5'-GGAGAAGAACATGGAATCAC-3'	5'-GTAATCTTGAACGCGGATATG-3'
OsTUBA1	5’-CATGATCTGCCAGTGTGGAGTT-3’	5’-GCCCATTAAGCCCCAAACAT-3’
OsActin	5’-TGCTATGTACGTCGC CATCCAG-3’	5’-AATGAGTAACCACGCTCCGTCA-3’

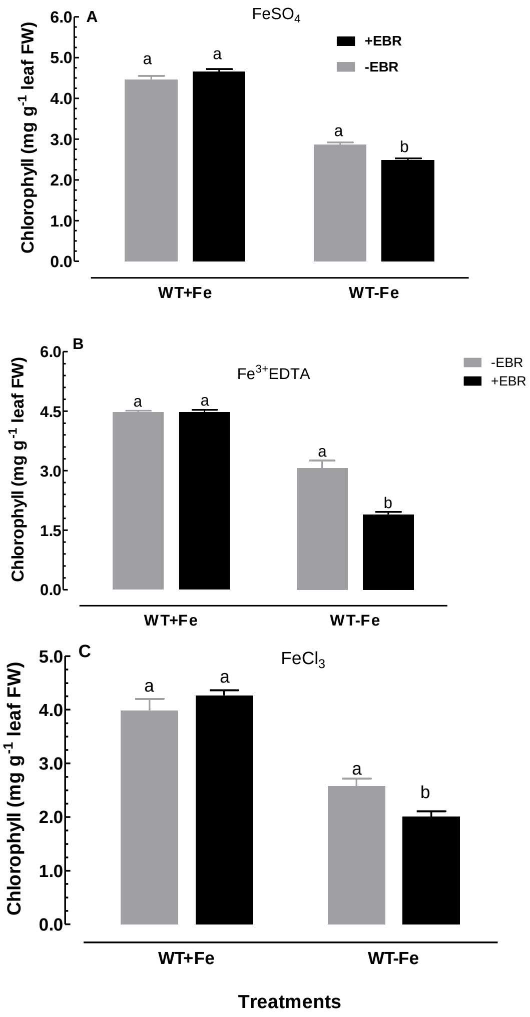

Supplementary Fig. S1. Effect of EBR on CHL in wild-type rice seedlings in the presence and absence of different forms of Fe (Fe^{3+}, Fe^{2+}). Photographs of 10-d-old WT rice seedlings exposed to $FeSO_4$ (A), Fe^{3+}EDTA (B), and $FeCl_3$ (C) sufficient or deficient medium with and without 100 nM EBR for 2 weeks. The chlorophyll content in WT rice leaves was determined (B). Data are means ± s.e. of six replicates. Means with different letters are significantly different ($P \leq 0.05$) with regard to the same Fe treatments.

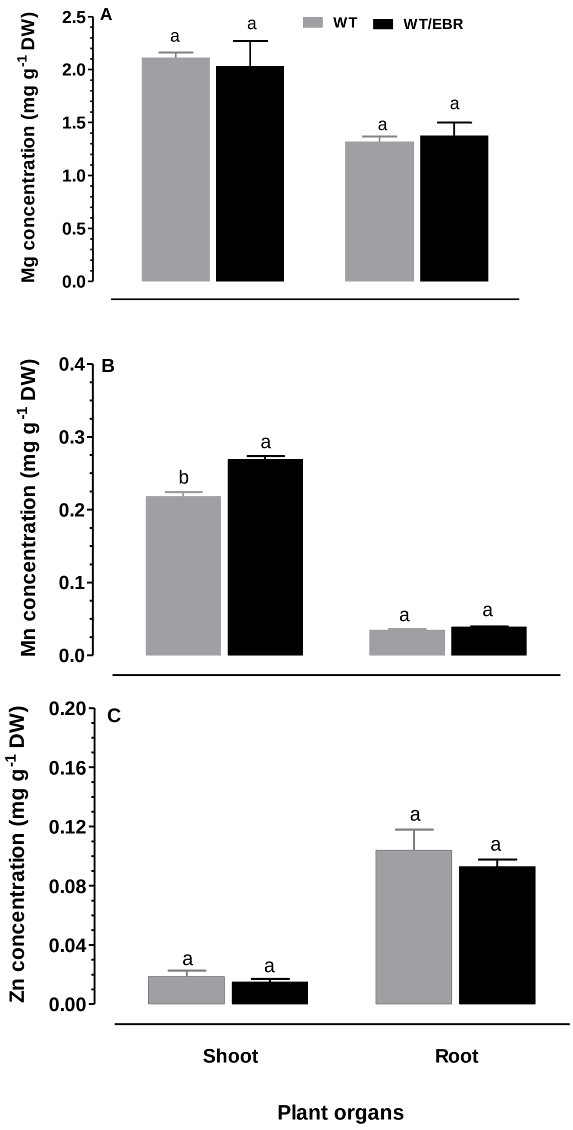

Supplementary Fig. S2. Effect of EBR on Mg, Mn and Zn concentrations in shoots and roots of wild-type rice seedlings (A, B, C). Ten-d-old WT seedlings were exposed to Fe-sufficient medium with and without 100 nM EBR for 2 weeks. After treatments, Mg, Mn and Zn concentrations were measured. Data are means ± s.e. (n = 4). Means with different letters are significantly different ($P \leqslant 0.05$) within the same Fe treatment.

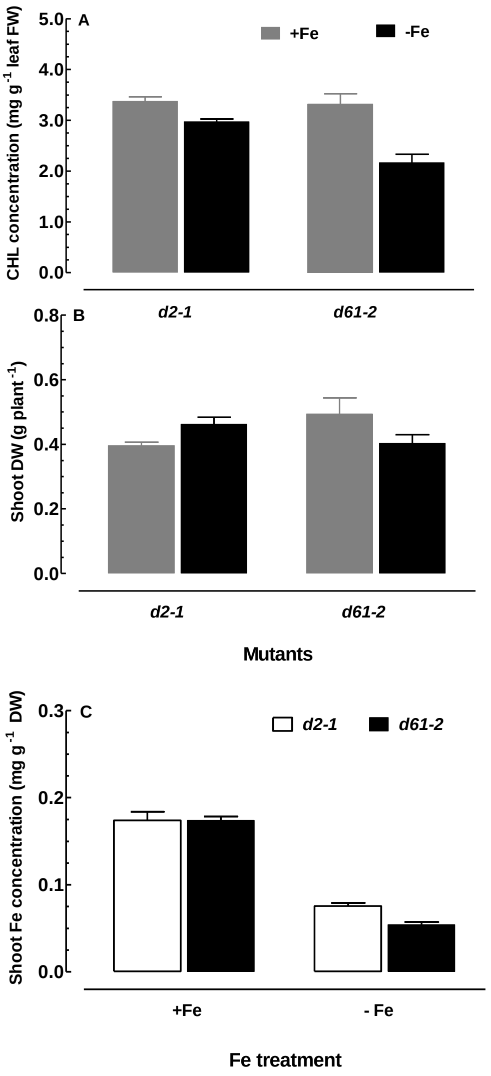

Supplemenray Fig. S3. Effect of Fe deficiency on CHL concentrations (A), shoot and root dry weight (B), Fe concentrations (C) in shoots of *d2-1* and *d61-2* mutants. Ten-d-old *d2-1* and *d61-2* (BR receptor mutant) seedlings were exposed to Fe-deficient medium for 2 weeks. After treatments, CHL, shoot DW and shoot Fe were measured. Data are means ± s.e. (n = 4).

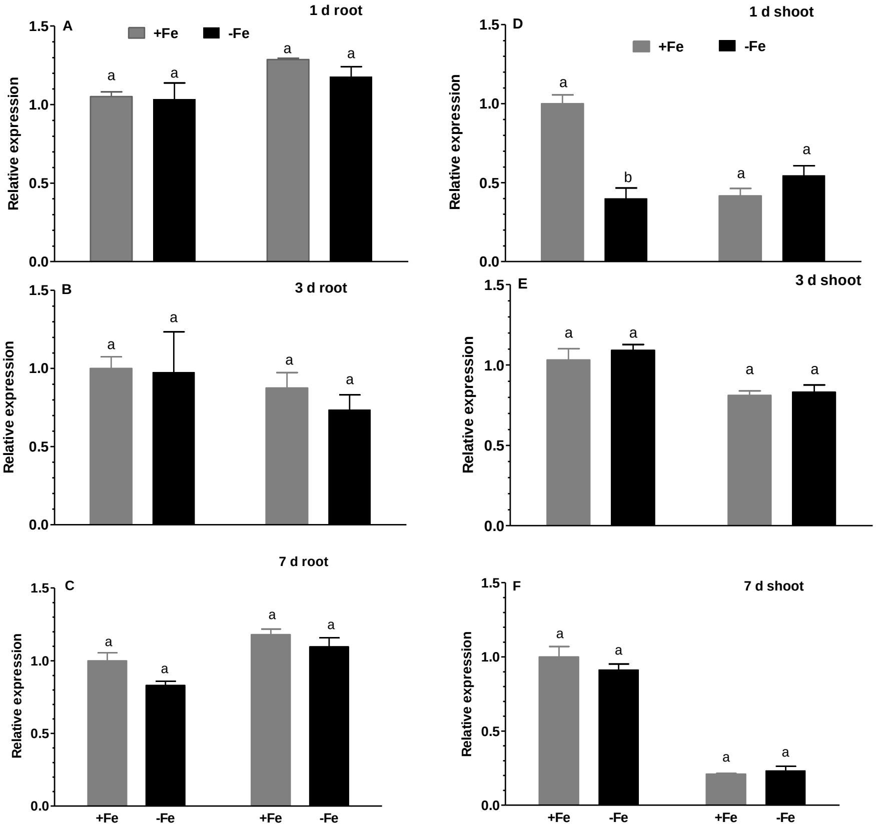

Supplementary Fig. S4. Time-course quantitative RT-PCR analysis of OsD2 in roots and shoots of WT and *d2-1* mutant plants under different Fe supply conditions. Data are means ± s.e. of three biological replicates. Means with different letters are significantly different within the same gene ($P \leqslant 0.05$)
